# Single cell transcriptome revealed tumor associated antigen (TAA) profile in lung adenocarcinoma (LUAD)

**DOI:** 10.1186/s40364-021-00287-8

**Published:** 2021-06-02

**Authors:** Fang Lv, Xueying Wu, Jin Song, Pan Wang, Shucheng Ren, Wei Guo, Qi Xue, Henghui Zhang, Jun Zhao

**Affiliations:** 1 Department of Thoracic Surgery, National Clinical Research Center for Cancer/Cancer Hospital, National Cancer Center, Chinese Academy of Medical Sciences and Peking Union Medical College, 100021 Beijing, China; 2 Beijing Shijitan Hospital, Ninth School of Clinical Medicine, School of Oncology, Capital Medical University, Peking University, Capital Medical University, 100038 Beijing, China; 3 Immupeutics Medicine Institute, Beijing, China; 4 Institute of Infectious Diseases, Beijing Ditan Hospital, Capital Medical University, Beijing, China

**Keywords:** Tumor associated antigens, Lung adenocarcinoma, Single‐cell RNA sequencing

## Abstract

It has been suggested that the heterogeneity of TAAs in lung cancer may affect the therapeutic response and disease progression. Up to now, several tumor-associated antigen (TAA)-based cancer vaccines have been investigated in lung adenocarcinoma (LUAD); however, most of them have failed at the stage of clinical trials. The present study suggests that inter-tumoral heterogeneity of TAAs is large at single-cell resolution, while the communication between tumor cells and infiltrating T cells is closely related to the expression profile of TAAs.

**Supplementary Information:**

The online version contains supplementary material available at 10.1186/s40364-021-00287-8.

To the Editor,

While non-small-cell lung cancer (NSCLC) ranks as the most commonly diagnosed malignancy worldwide, lung adenocarcinoma (LUAD) is the major histological subtype of NSCLC [1]. Although tumor-associated antigen (TAA)-based cancer vaccines have been used in NSCLC for a long time, their efficacy has reportedly been limited due to the deficient T cell priming [2]. Thus, further investigation of TAA expression and its interaction with T cells may provide telling evidence to support the application of TAA-based cancer vaccines in NSCLC. Here, we performed single-cell RNA sequencing (scRNA-seq) on primary lung tumor and distant metastases to profile the TAA diversity in LUAD.

We first merged scRNA-seq data from seven samples out of 6 patients with primary (*n* = 4) or metastatic (*n* = 2) LUAD (see Fig. 1a for patient attributes) using diagonalized canonical correlation analysis (CCA) to conduct a systematic comparison among the patients. Gene-expression profiles of 14,134 cells were retained after quality control filtering, and twenty clusters were identified using graph-based clustering on the informative principal-component analysis (PCA) space. As depicted in Figs. S1a and b, the clusters were largely consistent across patients in the integrated data. General cell types were annotated by reference to bulk transcriptomes (see “Method details”), and the annotation was supported by specific marker genes for matching cell types (Fig. S1c and d). Although the abundance of each cell type varied from one sample to another, most of immune and epithelial cell types were present in all patients (Fig. S1e). Epithelial cells (*n* = 3,442) were then subsetted and re-clustered. In agreement with previous reports [3–5], we found that the majority of epithelial cell (cancer cell) clusters were patient-specific (Fig. 1b). Strikingly, epithelial cells isolated from two primary lesions of the same patient showed a lesion-specific clustering.

**Fig. 1:**
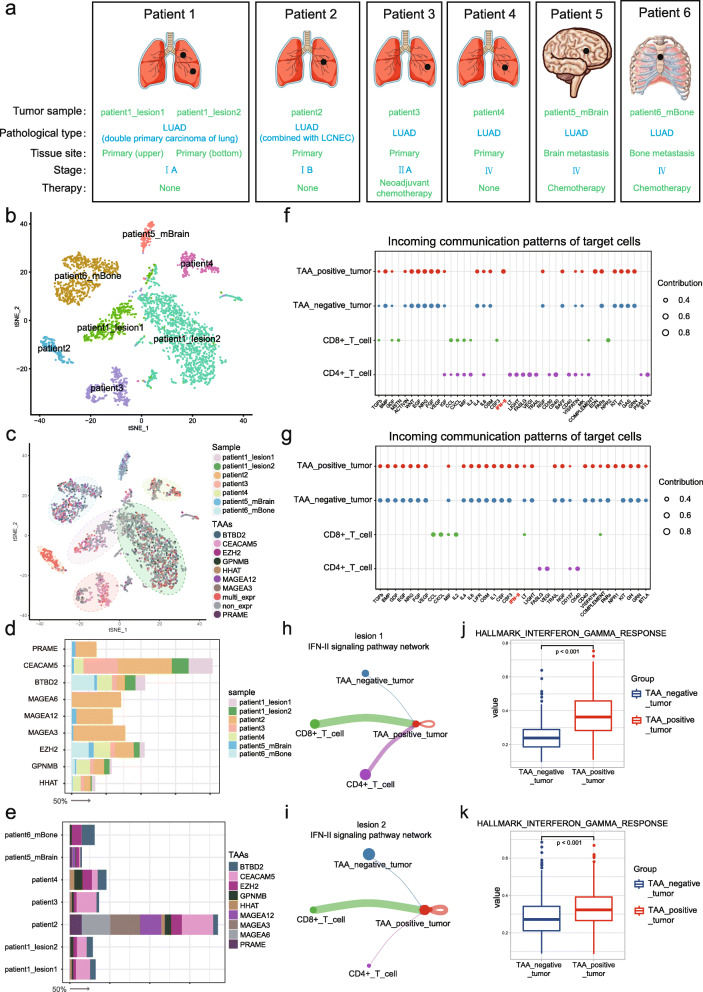
The TAA profile in lung adenocarcinoma. **a**A summary of the patient attributes. **b** and **c** t-SNE plot of 3,442 epithelial cells that were color-coded by the sample type of origin (**b**) or the TAA expression (**c**). **d** and **e** The fraction of cells that were colored by sample (**d**) or TAAs (**e**). In each image, the gray scale bar corresponds to 50 percent. **f **and** g** Dot plots showing the incoming signaling patterns of secreting cells in lesion 1 (**f**) or lesion 2 (**g**). The size of dots is in proportion to the communication probability. **h** and** i** Circle plots showing the inferred IFN-II signaling networks in lesion 1 (**h**) or lesion 2 (**i**). **j**and** k **Boxplots displaying the “ssgsea” scores of *HALLMARK INTERFERON GAMMA RESPONSE *in lesion 1 (**j**) or lesion 2 (**k**). *P*-values were calculated using the Wilcox-test. The box shows the upper and lower quartiles

Next, we collected information on the TAAs from the CTDatabase and ClinicalTrial.gov. For further analysis, we selected a total of seventy-five TAAs from TCGA database, which were frequently expressed in LUAD patients (Table S1). Among the 75 TAAs, 9 (*CEACAM5, BTBD2, EZH2, GPNMB, HHAT, PRAME, MAGEA3, MAGEA6, and MAGEA12*) were expressed at the single-cell level (log-normalized counts > 0) (Table S2; Fig. 1c). As illustrated in Fig. 1d, *CEACAM5* displayed the highest frequency of expression in early-stage LUAD (patient 1 lesion 1, 34.8 %; patient 1 lesion 2, 24.3 %; patient 2, 78.0 %; and patient 3, 49.6 %), while *EZH2, BTBD2* and *GPNMB* were among the most frequently expressed TAAs in stage IV primary tumor (23.8 %, 22.1 %, 20.4 %, respectively) and metastatic bone lesion (24.3 %, 32.5 %, 4.6 %, respectively). In the meantime, *MAGEA3, MAGEA6* and *MAGEA12* were detected only in patient 2 (LUAD combined with large cell neuroendocrine carcinoma) and patient 5 (brain metastases), suggestive of a large variation on the TAA profile across the different pathological types of tumor (Fig. 1e).

Lastly, we sought to determine whether cancer cells with TAA expression could potentiate T cell responses. The six patients included in this study were different in age, gender, race, pack-year smoking histories (above are environmental conditions), germline mutations or HLA typing (above are genetic background) which may cause significant interpatient immune-response variability. We therefore performed analyses in different lesions from patient 1 to avoid confounding effects of genetic background and environmental conditions. While T cell subset was classified into CD4^+^ (lesion 1, n = 442; lesion 2, n = 23) and CD8^+^ (lesion 1, n = 362; lesion 2, n = 295) T cells according to the cluster of differentiation receptors that were expressed in the cells, cancer cells were categorized as TAA-positive (lesion 1, n = 217; lesion 2, n = 639) or TAA-negative (lesion 1, n = 251; lesion 2, n = 797) cells based on whether or not the 75 TAAs from the database were expressed in the cells. As depicted in Fig. 1f g, relatively consistent incoming signaling patterns were present between TAA-positive and TAA-negative groups (see “Method details”). Surprisingly, the contribution score of IFN-II signaling reached statistical significance only in TAA-positive groups. Meanwhile, CD8^+^ T cells tended to send stronger IFN-II signal than CD4^+^ T cells (Fig. 1 h and 1i). Finally, a “ssgsea” score for quantifying the extent of IFN-γ pathway activation was calculated for each cancer cell (see “Method details”). As shown in Fig. 1j and k, TAA-positive cells in the two lesions exhibited a significantly higher score of IFN-γ response than the corresponding TAA-negative cells (both *p* < 0.001), suggesting that TAA-positive cancer cells may have a better response to antitumor immunity.

Collectively, this study suggests that while inter-tumoral heterogeneity of TAAs is large, the communication between tumor cells and infiltrating T cells is closely related to the TAA expression profile. Our findings might provide new clues for designing TAA-based cancer vaccines against LUAD.

## Supplementary Information

Additional file 1: Fig. S1. Integration and annotation of single-cell sequencing data.

Additional file 2: Table S1. Tumor-associated antigens (TAAs) expression frequency in TCGA database.

Additional file 3: Table S2. Tumor-associated antigens (TAAs) expression profiles.

Additional file 4: Method details.

## Data Availability

Raw data of patient 1 are available from the corresponding author upon reasonable request. Other supporting raw data can be found under Gene Expression Omnibus (GEO) number GSE123902 [6].

## Abbreviations

CCA: Canonical correlation analysis
CD: Cluster of differentiation
GEO: Gene Expression Omnibus
LCNEC: Large cell neuroendocrine carcinoma
LUAD: Lung adenocarcinoma
NSCLC: Non-small-cell lung cancer
PCA: Principal-component analysis
scRNAseq: Single-cell RNA sequencing
TAAs: Tumor associated antigens
TCGA: The Cancer Genome Atlas
t-SNE: T-distributed stochastic neighbor embedding
