# Supplementary material for: Single cell transcriptome revealed tumor associated antigen (TAA) profile in lung adenocarcinoma (LUAD)
Source: Biomark Res. 2021 Jun 2;9:41. doi: 10.1186/s40364-021-00287-8 (PMC8170805; doi:10.1186/s40364-021-00287-8)
Supplement: Supplementary file 1 — Integration and annotation of single-cell sequencing data. [file 40364_2021_287_MOESM1_ESM.docx]

**Additional file 1**

**Fig. S1**

**
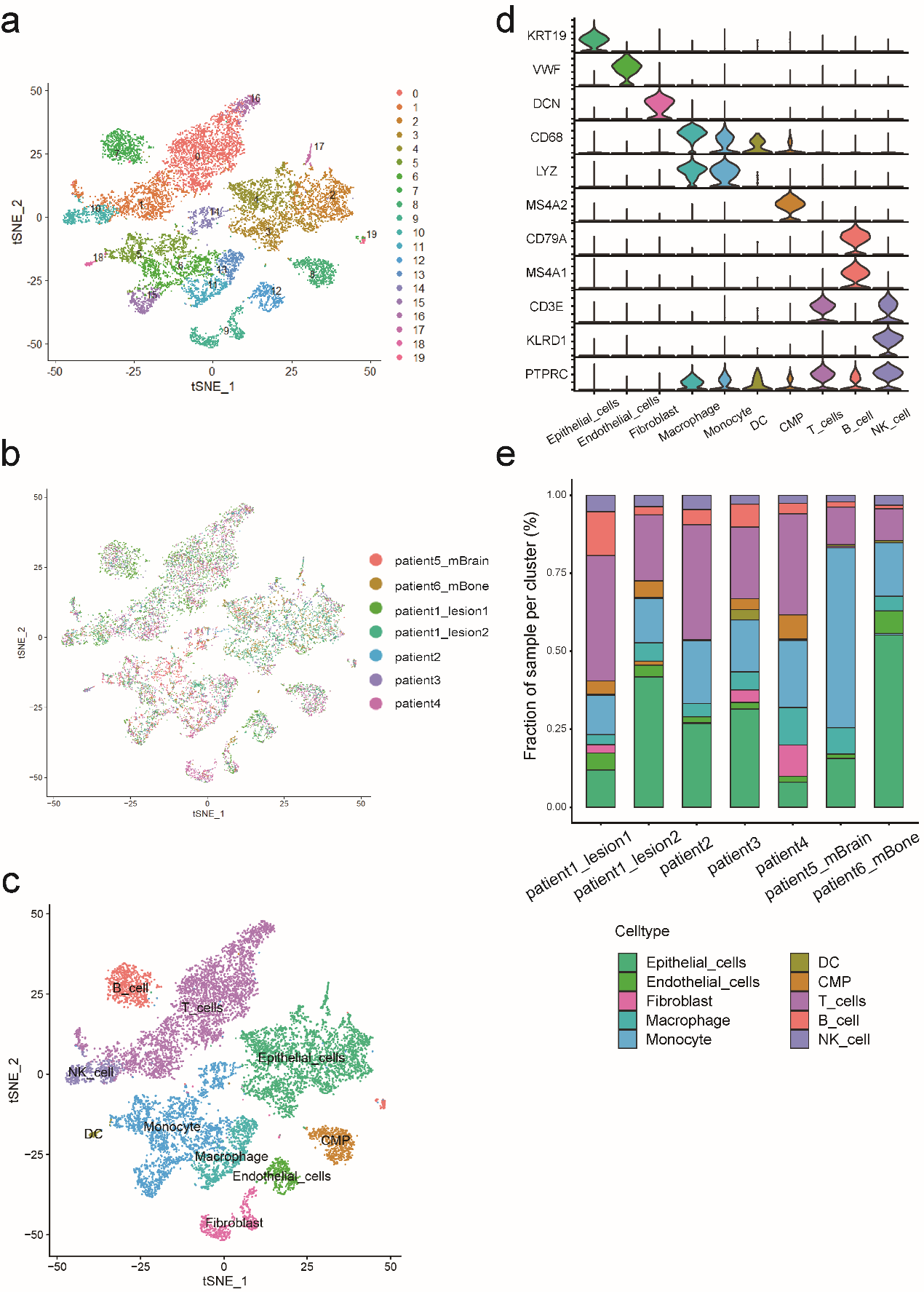
**

**Fig. S1 Integration and annotation of single-cell sequencing data.**

**a** T-distributed stochastic neighbor embedding (t-SNE) plot showing cell clusters. Each color represents a different cluster. **b** t-SNE plot of all cells colored by sample origin. **c** t-SNE plot of major classes of cells. **d** Violin plots showing the expression level distribution of specific marker genes. **e** Cell type fractions per sample.
