# Supplementary material for: Single cell transcriptome revealed tumor associated antigen (TAA) profile in lung adenocarcinoma (LUAD)
Source: Biomark Res. 2021 Jun 2;9:41. doi: 10.1186/s40364-021-00287-8 (PMC8170805; doi:10.1186/s40364-021-00287-8)
Supplement: Supplementary file 4 — Method details. [file 40364_2021_287_MOESM4_ESM.docx]

**Method details**

**Sample collection**

This study has been approved by Ethics Committees of National Cancer Center/National Clinical Research Center for Cancer/Cancer Hospital and we complied with all relevant ethical regulations.

One patient with untreated, non-metastatic, double primary lung tumors that underwent lung lobe resection with curative intent and that provided informed consent was included in this study. The remainder of the data were obtained from public database ([GSE123902](https://www.ncbi.nlm.nih.gov/geo/query/acc.cgi?acc=GSE123902)).

**Single-cell RNA sequencing**

Single-cell suspensions were converted to barcoded scRNA-seq libraries by using the Chromium Single Cell 5’ Library, Gel Bead & Multiplex Kit and Chip Kit (10x Genomics), aiming for an estimated 8,000 cells per library and following the manufacturer’s instructions. Libraries were sequenced on NovaSeq 6000, and mapped to the human genome (build GRCh38-3.0.0).

**Filtering and normalization of scRNA-seq data**

Raw gene expression matrices generated per sample using CellRanger (version 3.1.0) were combined in R (version 3.6.3), and converted to a Seurat object using the Seurat R package (version 3.1.5). Cells were further filtered with the following requirements: genes that were seen in at least three cells, cells should express at least 200 genes, cells that had unique molecular identifiers (UMIs) greater than 1000 and the mitochondrial gene expression less than 20%.

The filtered matrix was normalized using the Seurat function NormalizeData. Variable genes were found using the Seurat function FindVariableFeatures. Next, canonical correlation analysis (CCA) using the Seurat package was used to integrate scRNA-seq data among samples. The integrated data was scaled to regress out the sequencing depth for each cell. Variable genes that had been previously identified were used in principle component analysis (PCA) to reduce the dimensions of the data, and the first 30 principle components further summarized using t-distributed stochastic neighbor embedding (tSNE) dimensionality reduction.

**Analysis of cell-cell communication**

Cell-cell communication analysis was performed using the “CellChat” package (Version 0.0.1) in R. The majority of ligand–receptor interactions were curated on the basis of KEGG (Kyoto Encyclopedia of Genes and Genomes) signaling pathway database (<https://www.genome.jp/kegg/pathway.html>). The communication probability on signaling pathway level was computed using the function computeCommunProbPathway. The interactions with p-value < 0.05 are considered significant.

**Immune-related signature analysis**

The gene set for *HALLMARK_INTERFERON_GAMMA_RESPONSE* was obtained from the MSigDB database (<https://www.gsea-msigdb.org/gsea/msigdb/>). The signature score was calculated by single-sample gene set enrichment analysis using “GSVA” package (method = “ssgsea”) in R.

**Statistical analysis**

All statistical analyses were performed using R version 3.6.3 software (Institute for Statistics and Mathematics, Vienna, Austria; [www.r-project.org](http://www.r-project.org)). The Mann–Whitney U test was used for comparisons between two continuous variables. All differences with p < 0.05 were considered statistically significant
